# Editorial: Biological Strategies to Enhance the Anaerobic Digestion Performance: Fundamentals and Process Development

**DOI:** 10.3389/fmicb.2021.762875

**Published:** 2021-11-05

**Authors:** Shanfei Fu, Irini Angelidaki, Zeynep Cetecioglu, Qiang Kong, Yi Zheng, Panagiotis Tsapekos

**Affiliations:** ^1^School of Environmental and Civil Engineering, Jiangnan University, Wuxi, China; ^2^Department of Chemical and Biochemical Engineering, Technical University of Denmark, Kongens Lyngby, Denmark; ^3^Department of Chemical Engineering, Royal Institute of Technology, Stockholm, Sweden; ^4^College of Geography and Environment, Shandong Normal University, Jinan, China; ^5^Department of Grain Science and Industry, Kansas State University, Manhattan, KS, United States

**Keywords:** anaerobic digestion, biological pre-treatment, bioaugmentation, direct interspecies electron transfer, biogas plant surveillance

Anaerobic digestion (AD) is a well-recognized process for organic waste reduction, stabilization, and bioenergy recovery (Niu et al., 2021). During the AD process, organic substrate is converted into biogas through four main biological steps: hydrolysis, acidogenesis, acetogenesis, and methanogenesis (Wu et al., 2021). Biogas is the end-product of AD, which is considered an important renewable energy carrier. Though promising, some key challenges, such as low biogas production efficiency, long digestion period, limited volumetric efficiency, and high capital cost, still exist in the AD process. In addition to these challenges, the microbial compositions and synergy mechanisms in the AD systems could also be affected by many factors including operational parameters, ammonia, volatile fatty acids (VFAs) concentration, and salinity.

Within the AD process, steering microbiological processes could potentially be an economical, highly efficient, and environmentally friendly solution (Detman et al., 2021). Microbial resource management was introduced originally by Vestraete et al. as an efficient way to influence the outcome of the AD process by managing or altering the microbial community composition to achieve specific goals (Verstraete et al., 2007). Microbial community composition has been altered to mitigate ammonia inhibition, to achieve resilience and balance of the AD process, to avoid inhibition of specific toxicants, and to improve acetogenesis. Several approaches have been applied to microbial community management as a means of influencing microbial composition (Figure 1). Bioaugmentation is a method wherein specific microorganisms, either as pure cultures or as mixed cultures, have been added either once or over a period of time to increase the tolerance to ammonia (Yang et al., 2019) and lipid-rich substrate (Cirne et al., 2006) and enhance the biodegradation of lignocellulosic materials (Martin-Ryals et al., 2015; Tsapekos et al., 2017). Other biological strategies include enrichment and/or stimulation of special indigenous microorganisms to increase the competitiveness of these microorganisms by providing the preferred substrate or operating AD at specific conditions (e.g., temperature, pH, specific nutrients, and micro-aeration), which have been reported as promising methods to improve AD efficiency. However, further research is still needed to advance the AD process based on both fundamental understanding of the process and process engineering. Additionally, the studies on the variations of microbial communities and interactions among various microorganisms when the AD systems are subjected to certain external factors are also crucial to further unraveling the science of AD.

**Figure 1:**
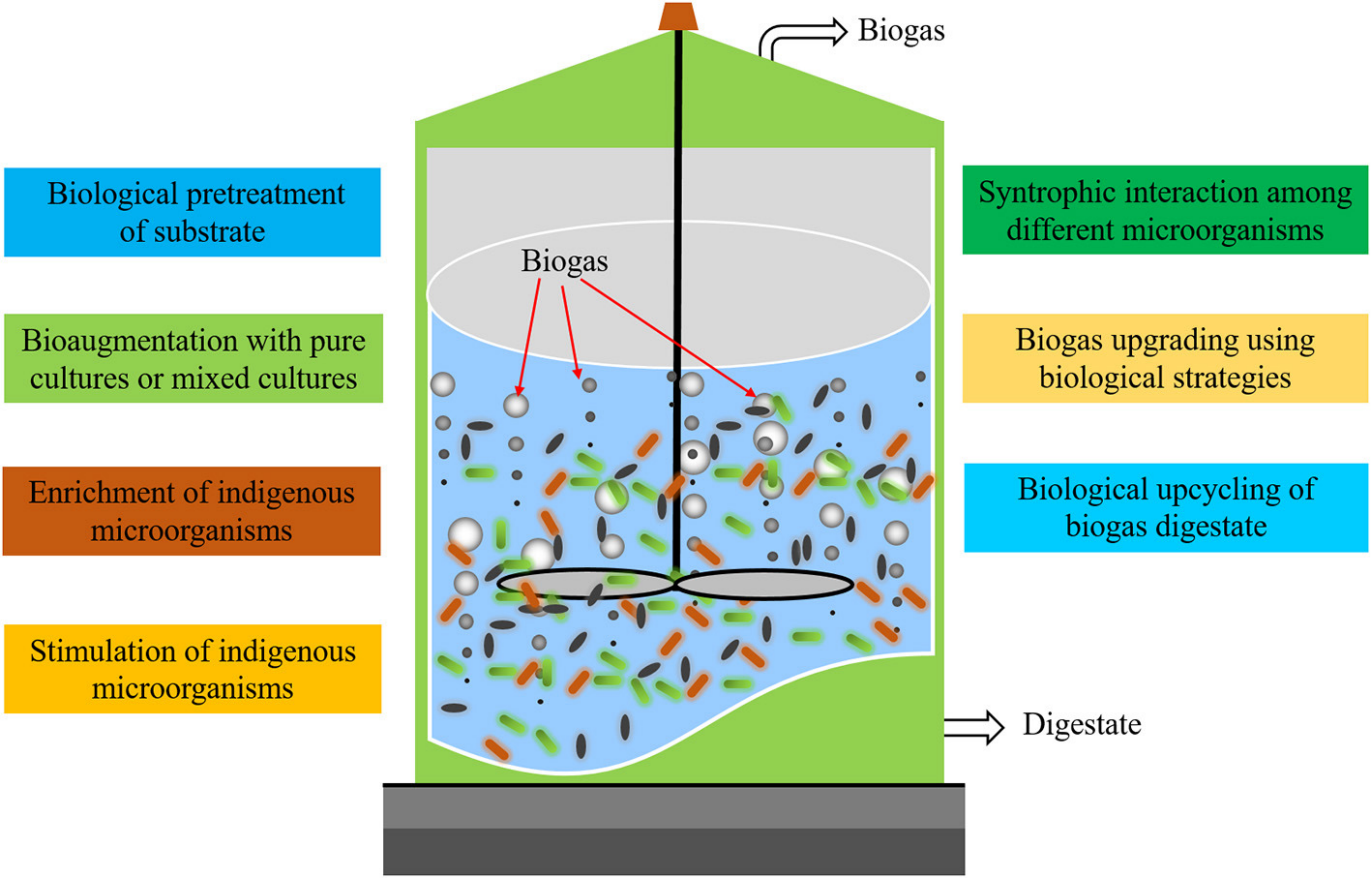
Biological strategies to enhance the anaerobic digestion performance.

This Research Topic aims to cover promising and novel research into biological strategies to enhance AD performance, surveillance of biogas plants, and promote AD commercialization. Song et al. tried to enhance the AD performance of paper waste by novel biological pre-treatment, which provides an efficient and environmentally friendly solution to low hydrolysis efficiency existing in AD of cellulosic substrate; Atasoy and Cetecioglu showed the bioaugmentation of AD to treat cheese production wastewater with *Clostridium aceticum* could significantly increase the total VFA production as well as acetic acid concentration in the VFA mixture. García Rea et al. focused on increasing the phenol conversion rate in anaerobic systems by adding high sodium, which promoted the enrichment of phenol degrader *Syntrophorhabdus* sp. and the acetoclastic methanogen *Methanosaeta* sp.; Logroño et al. reported inoculum enriched in *Methanobacterium* genus can have functional resilience in terms of hydrogen consumption and methane production upon starvation periods. Xu et al. reported that a hydrothermal pre-treatment (HPT) combined with the addition of hydrochar could promote the actual biogas yield of anaerobic digestion of dead pig carcasses, since HPT considerably increased lipid decomposition and, to a lesser extent, proteins, and that hydrochar reduced ammonia inhibition by different mechanisms; Ceron-Chafla et al. showed the limited propionate conversion at elevated pCO_2_ could be promoted by alternative and more resilient syntrophic propionate oxidizing bacteria and building up biomass adaptation to environmental conditions via directional selection of microbial community. Zhang et al. found that *Lactobacillus plantarum* QZ227 was a suitable silage additive to accumulate lactic acid and protect lignocellulosic biomass from contamination under freezing and thawing at low temperatures; Pang et al. demonstrated that the protease activity, protein-degrading bacteria, and acidogens were improved, while the α-glucosidase activity and the carbohydrate-degrading bacteria were inhibited under high salinity during AD. Singh et al. developed a strategy wherein a high-throughput microbiological surveillance approach was used to visualize the potential acetogenic population in commercial biogas digesters by using formyltetrahydrofolate synthetase (FTHFS) gene amplicons and unsupervised data analysis with the AcetoScan pipeline, which could assist in management of stable AD operations.

Overall, the published articles revealed biologically-based strategies to overcome barriers existing in anaerobic environments and enhance AD process performance. Though many papers are published on this topic every year, and the subject is attracting increasing attention, breakthroughs are still limited. More efforts should be made to reveal and understand fundamental mechanisms using advanced technologies (e.g., genome-centric metatranscriptomics, isotope tracing techniques, high-throughput sequencing, etc.). Subsequently, the generated knowledge can pave the way for the construction of highly efficient AD systems.

## Author Contributions

All authors listed have made a substantial, direct and intellectual contribution to the work, and approved it for publication.

## Conflict of Interest

The authors declare that the research was conducted in the absence of any commercial or financial relationships that could be construed as a potential conflict of interest.

## Publisher's Note

All claims expressed in this article are solely those of the authors and do not necessarily represent those of their affiliated organizations, or those of the publisher, the editors and the reviewers. Any product that may be evaluated in this article, or claim that may be made by its manufacturer, is not guaranteed or endorsed by the publisher.
